# Untangling the complexity of opioid receptor function

**DOI:** 10.1038/s41386-018-0225-3

**Published:** 2018-09-24

**Authors:** Rita J. Valentino, Nora D. Volkow

**Affiliations:** The National Institute on Drug Abuse, Bethesda, MD 20892 USA

## Abstract

Mu opioid receptor agonists are among the most powerful analgesic medications but also among the most addictive. The current opioid crisis has energized a quest to develop opioid analgesics that are devoid of untoward effects. Since their discovery in the 1970’s, there have been major advances in our understanding of the endogenous opioid systems that these drugs target. Yet many questions remain and the development of non-addictive opioid analgesics has not been achieved. However, access to new molecular, genetic and computational tools have begun to elucidate the structural dynamics of opioid receptors, the scaffolding that links them to intracellular signaling cascades, their cellular trafficking and the distinct ways that various opioid drugs modify them. This mini-review highlights some of the chemical and pharmacological findings and new perspectives that have arisen from studies using these tools. They reveal multiple layers of complexity of opioid receptor function, including a spatiotemporal specificity in opioid receptor-induced cellular signaling, ligand-directed biased signaling, allosteric modulation of ligand interactions, heterodimerization of different opioid receptors, and the existence of slice variants with different ligand specificity. By untangling these layers, basic research into the chemistry and pharmacology of opioid receptors is guiding the way towards deciphering the mysteries of tolerance and physical dependence that have plagued the field and is providing a platform for the development of more effective and safer opioids.

The 1970’s heralded a new era in the opioid field with the discovery that opiate drugs produce their effects by binding to specific binding sites in brain, followed by the discovery that brain neurons synthesize opioid-like peptides that produce similar effects through actions at the same receptors [1–4]. Coupled with the findings that naloxone-reversible analgesia could be produced by stimulation of specific brain regions, this solidified the transformative idea that opiates act by mimicking the endogenous opioid systems [5]. Gene cloning and brain mapping revealed three opioid peptide systems encoded by individual genes for pre-proenkephalin, pre-proopiomelanocortin, and pre-prodynorphin that have distinct brain distributions [6–12]. Likewise, three distinct receptors were cloned, μ (MOR), κ (KOR), and δ (DOR), with different selectivities for the individual endogenous peptides and for the various opiate drugs used pharmacologically [13–15].

Opioid receptor localization, first based on receptor binding, then on in situ hybridization and more recently on the localization of fluorescently tagged receptors in genetically modified mice show similar distinct but overlapping distributions for the three receptors [16–20] (Fig. 1). Taken with reports of the cellular and circuit responses to receptor activation, these findings are shaping our evolving understanding of how different opioids produce their rewarding or dysphoric effects, pro- and anti-stress effects, cognitive effects that govern decisions, and their analgesic and respiratory depressing effects (see for review [21, 22]. For example, rewarding effects of MOR activation have long been considered to be mediated by inhibition of GABA interneurons in the ventral tegmental area (VTA) resulting in disinhibition of dopamine neurons [23, 24]. However, recent evidence revealed that this is primarily due to inhibition of a potent GABA input from the rostromedial tegmental nucleus and to a lesser extent, inhibition of a GABA input from the nucleus accumbens (NAC)-D2 expressing neurons [25]. Similar MOR-induced inhibition of GABA neurons in the periaqueductal gray (PAG) and raphe magnus may contribute to analgesia [26, 27]. Inhibition of GABA interneurons in the hippocampus by MOR and DOR agonists increases pyramidal cell activity, an effect that could facilitate learning and memory associated with drug taking [28, 29]. The well-characterized direct inhibition of norepinephrine locus coeruleus (LC) neurons by MOR tones down the activation of this central stress response system and promotes stress recovery [30, 31]. In contrast, robust activation of LC neurons associated with opioid withdrawal may underlie the hyperarousal and sleep disturbance that interferes with recovery [32–34]. Notably, the habenula, a brain region of especially high MOR density, is central to a circuit that mediates aversion and opposes reward through its inhibition of dopamine VTA neurons [35, 36]. Activation of MOR in the lateral habenula has mixed excitatory and inhibitory effects and effects in the medial habenula are not yet well characterized [37, 38]. KORs are prominently localized to axon terminals in many brain regions and presynaptically inhibit neurotransmitter release. KOR-related inhibition of dopamine release in both the NAC and PFC is associated with aversion and can oppose the effects of MOR [39, 40]. KOR-mediated presynaptic inhibition of glutamate release from axon terminals in the NAC may restrain responses to stimuli and generally decrease motivation. Similar presynaptic effects have been described in the LC where KOR activation blunts the typical robust excitation elicited by salient sensory stimuli that is glutamate mediated, as well as LC activation by a stressor mediated by corticotropin-releasing factor inputs [41]. By decreasing the typical arousal response to these stimuli, KOR regulation of LC afferents can contribute to flattened motivation and affect.

**Fig. 1:**
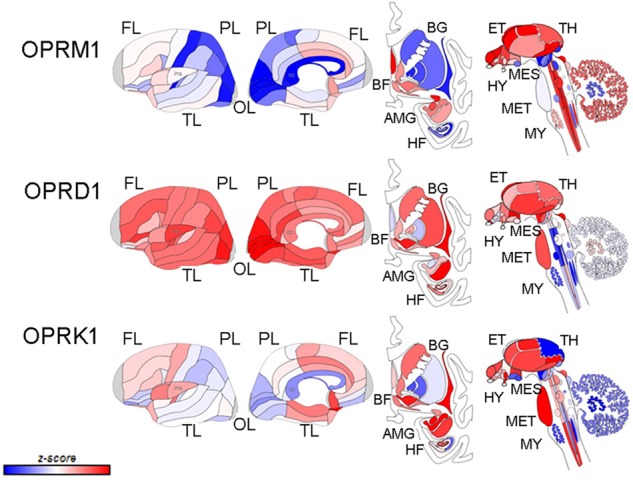
Schematic depicting the differential localization of MOR, DOR, and KOR based on gene expression patterns in human brain (donor H0351,1016, 55 years., Male White or Caucasian from the Allen Brain Atlas http://www.brain-map.org). The first two columns show outer and inner surfaces of the left hemisphere. Subcortical structures are represented from the frontal view (third column), and subcortical and brainstem structures are shown in the side view (fourth column). The color bar displays expression values using *z*-score normalization. (Modified with permission from; [42] http://creativecommons.org/licenses/by/4.0/). The same image from [42] was recreated using the Allen Brain Atlas and labels were added

These examples and others underscore how research on the functions of the three opioid signaling systems is revealing unique and counterbalancing roles as they relate to their regulation of pain, stress, and affect [21] (Fig. 2). Though the MOR is the main target for opioid analgesics, the DOR and KOR also regulate pain and analgesia and the relative affinities of opioid analgesics for these receptors confers them unique properties. The rewarding effects of opioids also rely on the MOR, though DOR and KOR modulate them through the regulation of hedonics, mood, and stress reactivity (Fig. 2) [21]. Specifically, while MOR agonists produce euphoria and promote stress coping, KOR agonists produce dysphoria, stress-like responses and negative affect, while agonists at DOR reduce anxiety and promote positive affect. The multiplicity of opioid receptors inspired the design of agonists and antagonists with different potencies, efficacies and selectivities for MOR, DOR, and KOR based on structure activity relationships and with different pharmacokinetics in an effort to develop analgesics with less adverse effects. These are also being pursued as potential treatments for addiction and depression [42]. Although promising, this strategy has yet to yield a potent opioid analgesic that is not rewarding, lacks tolerance, does not trigger physical dependence or produce respiratory depression.

**Fig. 2:**
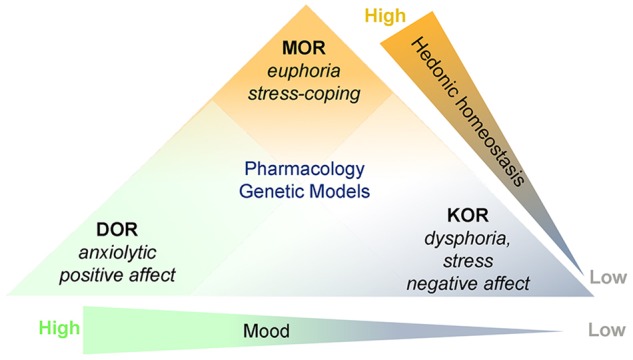
Schematic depicting that although agonists at MOR, KOR, and DOR are all analgesic, pharmacological studies, and genetic models reveal that they are at different ends of mood and hedonic continuums. MOR agonists produce euphoria and promote stress coping. At the other end of the hedonic continuum, KOR agonists produce dysphoria and are associated with stress and negative affect. DOR is on the opposite end of the continuum describing mood and DOR agonists have anxiolytic and antidepressant activity. This figure was revised with permission (Fig. 1, [81])

New tools and innovative approaches are revealing that opioid receptors are more complex than previously appreciated and this can account for past difficulties in designing ideal ligands. Here we describe studies using state-of-the-art approaches to reveal the structural nuances of opioid receptors in different conformational states and of computational approaches to design drugs based on this information. We highlight the power of new tools for visualizing receptor localization and activation of signaling cascades at an ultrastructural level that are challenging conventional views of how opioid receptors signal and the significance of receptor trafficking. These studies are shaping a new perspective of opioid receptor chemistry and pharmacology that will ultimately result in optimal drug design.

The intent of this review is to highlight new perspectives on opioid receptor structure and signaling. A review of the cellular consequences of opioid receptor activation was considered to extend beyond the scope of this review. Notably, this is described in detail in Williams et al. [22].

## Structural dynamics of the opioid receptors

Delineating the crystal structures of opioid receptors has been pivotal for understanding receptor function and providing new avenues for drug design. The crystal structures of the MOR, DOR, and KOR in the inactive conformation (i.e., bound to antagonists) identified the spatial organization of key molecules within the receptor that make up the binding pocket and determine ligand selectivity [43–45]. The elucidation of the structural dynamics that determine the transduction of receptor binding to signaling required the challenging task of determining their crystal structure in their less stable, active conformation. A major advance that facilitated this involved the use of nanobodies, which are single chain antibodies that mimic the G-proteins that couple to and stabilize receptors in an active conformation. To date, the crystal structures of MOR and KOR have been identified in the active conformation using this approach [46, 47]. Additionally, this approach was used with cryo-electron microscopy to identify the active conformation of the MOR bound to the peptide agonist [48]. Knowledge of the receptor conformations in active vs. inactive states facilitates computational approaches for high throughput drug design. By computationally docking large molecular libraries with identified receptor structures, new ligands with predicted potency, efficacy, and selectivity can be developed. For example, using this approach, a MOR ligand (PZM21) with potent Gi activation and low β-arrestin recruitment (biased agonist) was identified that lacked respiratory depression and appeared to have less reinforcing effects at doses that were equi-analgesic with morphine [49] and is being explored as a strategy for the development of safer MOR opioid agonists [50]. Similar approaches have identified Gi-biased agonists for the KOR, which have analgesic efficacy and lack dysphoria and potent antagonists, which also have potential as therapeutics for treatment of addiction and as antidepressants [51].

## Allosteric regulation

Allosteric binding sites offer a therapeutic target for modulation of opioid receptor activity [52, 53]. These sites are spatially separated from the orthostatic site or ligand binding pocket and they modulate receptor function either positively (positive allosteric modulators, PAMs) or negatively (negative allosteric modulators, NAMs). These could potentially be used to lower the analgesic dose of opioid agonists, although it is not clear whether this approach would eliminate tolerance and dependence or other adverse effects associated with opioid administration. An appealing concept is that PAMs could potentiate the effects of endogenous opioids that are released during pain, which would restrict analgesia both temporally and spatially. The use of PAMs to potentiate placebo-induced analgesia may have therapeutic rationale given the proposed role for endogenous opioids in the placebo response [54]. Notably, there is evidence for NAM activity of THC, cannabidiol, and Salvinorin A at the MOR [55, 56]. A site that accounts for the allosteric modulation of the DOR by sodium has been structurally characterized [57]. However, the structural identification of allosteric sites for other modulators or at other opioid receptors has lagged because of the difficulties inherent in crystallization of the receptors; though cryo-electron microscopy, which does not require crystallization, has accelerated the rate at which receptor structures are being identified and may resolve this question. Molecular docking and computational methods are providing information about the structure and location of the allosteric site with respect to the orthostatic site and this will facilitate the development of this class of compounds.

## Biased signaling

**Fig. 3:**
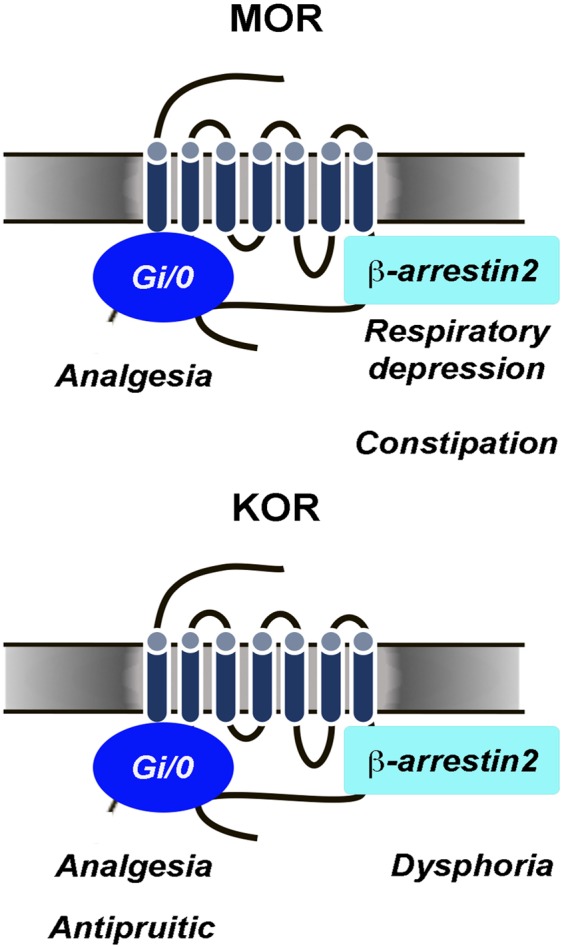
Differential consequences of MOR and KOR biased signaling. For the MOR, analgesic effects are proposed to occur through Gi/o-protein-dependent signaling whereas respiratory depression and constipation are proposed to occur through β-arrestin 2-related signaling. For the KOR analgesia and antipruitic effects are proposed to occur through Gi/o-protein-dependent signaling and dysphoric effects associated with KOR are proposed to occur through β-arrestin 2-related signaling

Perhaps one of the most promising concepts to emerge in the last decade is that of ligand-dependent biased signaling whereby the same receptor can engage either G-protein-dependent signaling pathways or β-arrestin-dependent signaling depending on the ligand bound. If differential signaling produces distinct consequences, this feature can be used to custom design drugs for desired effects. For example, it has been proposed that analgesic and antipruritic actions of KORs are associated with Gi-protein signaling, whereas the dysphoric effects are associated with β-arrestin [58, 59]. Likewise, MOR-induced analgesia has been associated with Gi-dependent signaling, whereas certain adverse effects including respiratory depression and constipation have been proposed to be mediated by β-arrestin (Fig. 3). This was originally based on findings using β-arrestin-2 knockout mice which show enhanced analgesia produced by morphine with less tolerance, respiratory depression and constipation [60–62]. Consistent with this, the compound PZM21 that was identified by computational approaches as described above, is a Gi-protein biased compound that has these features [49]. This research has led to the idea of a bias factor, the ratio of some endpoint of Gi-signaling (typically GTPγS binding) to β-arrestin recruitment that may predict the therapeutic window for analgesia versus respiratory depression [50, 63]. This strategy is leading one avenue of therapeutic development. Currently, TRV130, a highly Gi-biased MOR agonist that showed promise in preclinical studies is undergoing Phase 3 clinical trials for the treatment of moderate to severe pain (NCT02820324) [64].

To date, little is known regarding the structural features that confer bias signaling. Biased ligands must stabilize a conformation that favors association with either the G-protein or β-arrestin. For the DOR, the sodium allosteric site has been linked to β-arrestin bias because mutations in that site that decrease sodium binding conversely augment β-arrestin recruitment [57]. Structural features that confer Gi-protein bias to the KOR have also been identified by comparing the results of docking IBNtxA with the MOR, where it exhibits a Gi-protein bias, to docking with the KOR, where it is unbiased [47]. If the development of biased opioid agonists proves to be a major therapeutic advance, an important challenge will be to identify structural determinants of different degrees of bias. This will allow for high throughput design of drugs with particular bias indices and therapeutic windows.

## Receptor heteromerization

Evidence that opioid receptors form and can function as heteromers suggests another layer of complexity and another route for manipulating opioid receptor function. Functional interactions between opioid receptors first suggested the existence of opioid receptor heterodimers. This was supported by more direct evidence including co-immunoprecipitation of the two receptors and proximity in cells as determined by fluorescence resonance energy transfer (FRET) or bioluminence resonance energy transfer (BRET) (see for review [65]. Heterodimers composed of different opioid receptors (e.g., DOR-MOR, DOR-KOR, and MOR-KOR) or between opioid and non-opioid receptors (e.g., DOR-CB1) have been reported but the DOR-MOR heterodimer is the most well studied. DOR-MOR heteromers have been co-immunoprecipitated from spinal cord of wildtype but not DOR knockout mice [66]. Ligand binding to one protomer of DOR-MOR acts like an allosteric regulator to increase the affinity of a ligand to the partner protomer [66]. Interestingly, there is evidence for unconventional signaling by DOR-MOR, including coupling to Gz and enhancing calcium signaling [67, 68]. Individual partners can traffic independently or as the heteromer depending on the agonist bound. Notably, bivalent ligands that bridge the two receptors can hinder internalization, a concept that may be taken advantage of therapeutically. One therapeutic lead has been the development of a bivalent ligands that consist of a MOR agonist (oxymorphone) and DOR antagonist (naltrindole) separated by different spacer lengths. These have shown analgesic activity with comparatively decreased rewarding effects, and diminished development of tolerance and dependence [69, 70].

Heterodimers composed of MOR and the nociceptin/orphanin F/Q receptor (NOPR) have been co-immunoprecipitated from rat dorsal root ganglia, as well as heterologous cells [71]. Although agonists have higher binding affinity to the heteromer compared to the individual receptors, MOR-related signaling is selectively reduced, an effect that could have therapeutic benefit. An agonist that is thought to act by binding to this heteromer (IBNtx-A) showed potent analgesic effects in the absence of respiratory depression, constipation, rewarding effects or development of physical dependence [72].

Another heteromer of potential therapeutic interest is that between MOR and the galanin receptor subtype, Gal1 (Gal1R), since galanin counteracts the effects of MOR agonists. In the brain, these two receptors co-localize in the ventral tegmental area (VTA), a brain region that is central to drug reward. The MOR-GAL1R heteromer was shown to mediate galanin’s inhibition of DA neuron activation in VTA by MOR agonists; and thus, bivalent molecules that target this heteromer could potentially lead to the development of MOR analgesics devoid of rewarding effects [73].

The existence of functional opioid receptor heteromers remains controversial because these are difficult to study and most evidence relies on the use of heterologous cells and on antibodies that have limitations with regard to specificity. Convergent evidence in support of the existence of opioid receptor heteromers include detection in the spinal cord and dorsal root ganglia, disruption of co-immunoprecipitation by expressing one receptor with alterations of the C-terminus and activity of bivalent agonists that is absent when the heteromer is disrupted or blocked by antibodies. Interestingly, there is structural evidence from x-ray crystallography supporting the existence of MOR homodimers although the possibility that this was an artifact of crystallization could not be ruled out [44].

To circumvent the limitations of antibodies, double mutant mice expressing fluorescently tagged MOR and DOR receptors have been generated and co-localization of MOR and DOR examined [16, 74]. Studies using this model suggested that co-localization of the receptors was minimal and mostly limited to spinal cord and the dorsal root ganglia. However, one study showed co-localization in hippocampus, hypothalamus, lateral parabrachial nucleus, and certain brainstem regions [16]. This study found evidence from co-immunoprecipitation for proximity of the receptors that could support heterodimerization in the hippocampus. Given their potential as novel therapeutic targets, the systematic investigation of the in vivo existence and function of opioid heteromers with better tools is warranted.

## Truncated receptors, splice variants

The cloning of MOR uncovered the complexity of its gene *OPRM1* and the existence of multiple splice variants. Some of these variants are truncated and do not have traditional G-protein coupled receptors structures [75]. Specifically, *OPRM1*, contains two independent promoters; the exon 1 (E1) and the exon 11 (E11) promoters, that generate multiple variants. The E1 promoter generates seven transmembrane domain G protein-coupled receptor variants, whereas the E11 promoter generates truncated six transmembrane domain receptors. Genetic models in which the splice variants are deleted are revealing the functional relevance of different components of the receptor [75, 76]. These studies demonstrate that variants can influence the degree of tolerance, physical dependence and reward and the degree of signaling bias of certain agonists. For example, the 7TM variants are essential for morphine analgesia whereas the 6TM variants are not [77] although 6TM variants are important for analgesic effects of other MOR agonists [78] in models of thermal, neuropathic, and inflammatory pain without producing respiratory depression, physical dependence, or reward [79]. This line of research could explain individual variabilities in opioid responses and be a basis for individualized therapy.

## Ligand-specific spatiotemporal organization of opioid receptor signaling

As for other G-protein coupled receptors, MOR-initiated signaling has been thought to occur solely at the plasma membrane. MOR activation is followed by β-arrestin recruitment and internalization into endosomes from where MOR is thought to be either recycled to the plasma membrane or shuttled to lysosomes for degradation. This conventional model was recently upended by a transformative study demonstrating that depending on the ligand, MOR activation can occur not only at the plasma membrane, but within different cytoplasmic compartments [80]. To demonstrate this, MOR was selectively visualized in its activated state using a conformationally specific fluorescent-tagged nanobody. Both, peptide agonists (DAMGO) and alkaloids, such as morphine produced a rapid and short-lived signal at the plasma membrane indicative of MOR activation. Following exposure to the opioid peptide, MOR was detected in the active state in endosomes, peaking by 20 min. This could be reversed by naloxone but not by a membrane impermeant opioid antagonist. Importantly, there was evidence of Gi-related signaling within these endosomes with the same time course, indicating that endosomal MOR signaling contributes to the overall cellular signaling initiated by agonist binding. In contrast to opioid peptides, morphine, and etorphine cross the plasma membrane where they activate MOR located on the Golgi apparatus, a process that takes on the order of tens of seconds. This study changed the landscape of MOR function by demonstrating that receptor signaling occurs at unconventional cytoplasmic compartments with a spatial and temporal specificity that is dependent on the ligand bound. Critical questions raised by these findings relate to the nature of the signals initiated in these distinct cellular compartments and their downstream contribution to either therapeutic or pathological effects of opioids. It is also important to determine the profile of spatiotemporal aspects of MOR signaling initiated by different opioid agonists and a corresponding profile for different antagonists. Together, this information can be used to design more specific therapeutics. Importantly, the spatial organization of MOR-related signaling and the distinctions between different agonists may hold the keys to understanding the mechanisms by which opioids produce tolerance and dependence.

## Translating advances in opioid receptor research to the opioid crisis

Here we described how the development of new tools and approaches advanced our knowledge of opioid receptor function. Nanobody technology coupled with innovations in microscopy are providing high resolution maps of receptor structure, which with high throughput computation, can be used to hasten the development of novel opioids that lack adverse effects. The same technology is providing a window through which opioid receptor activation and trafficking between cellular compartments can be visualized. This is reframing our perspective of the cellular consequences of agonist binding to opioid receptors and revealing novel cellular mechanisms that can be targeted. Advances in genetics are identifying granular distinctions in receptors that could be a basis for understanding individual differences in vulnerabilities. Genetic models and tools that allow manipulation of receptor levels and activity reveal important information on the distinct functions of the different opioid receptors. Similarly, the β-arrestin knockout mouse is an example of a genetic model that has been pivotal in the concept of biased opioid receptor signaling. Though many scientific questions still remain unresolved (Table 1), the new advances, by revealing molecular and cellular fundamentals of opioid receptor function, bring us closer to understanding the mechanisms by which opioids produce tolerance, physical dependence and addiction and towards developing a rational therapeutic design of safe, effective opioid analgesics.

**Table 1:** Questions to guide future research on opioids and pain

*Molecular/cellular*
Identification and characterization of intracellular opioid signaling and its function
Mechanisms that restrict tolerance to signaling by endogenous opioids as distinct from tolerance and physical dependence associated with opioid drugs
Cellular neuroadaptations to chronic opioid signaling (e.g., cAMP, beta-arrestin)
Role of opioid receptor heteromers in analgesia, tolerance, and reward
*Systems/neurocircuitry*
Role of the endogenous opioid systems in the transition from acute to chronic pain
Interaction between pain and opioid reward and addiction
Overlap between opioid-mediated affect and opioid-induced analgesia
Mechanisms underlying co-morbidity of opioid addiction with pain and depression and of pain with depression
Role of endogenous opioids in the beneficial effects of sleep on pain
Sex differences in the endogenous opioid system that might underlie greater vulnerability of females to chronic pain syndromes and to depression
Role of the endogenous opioid sytem in social bonding
Role of the endogenous opioid system on sleep
Effects of aging on endogenous opioid function
*Therapeutics*
Engagement of peripheral versus central opioid receptor signaling for the management of pain
Bivalent ligands as targets for analgesia and treatment of opioid addiction
Targeting synthesis and degradation of endogenous opioid peptides as potential analgesics
Drug combinations to minimize tolerance and physical dependence
Individualizing treatments based on factors such as sex, age, genetics, and comorbidities
